# Negotiation of Sleepwalking in the Age of Algorithms: Agency, Interpretation and Everyday Adaptations

**DOI:** 10.1111/1467-9566.70238

**Published:** 2026-07-15

**Authors:** Svenja Reinhardt, Nico Wettmann

**Affiliations:** ^1^ Institute for the History of Medicine and Science Studies University of Lübeck Lübeck Germany; ^2^ Institute of Sociology Philipps University of Marburg Marburg Germany

## Abstract

Digital technologies, including sleep‐tracking devices and smart mattresses, are becoming increasingly prevalent for gaining certainty and insight into unconscious behaviours such as sleepwalking. Sleepwalking, also known as somnambulism, is difficult to diagnose because it is episodic and unpredictable. Drawing on ethnographic research in sleep medical facilities and online communities focused on sleep tracking, this study examines how algorithmic systems shape our understanding of sleepwalking and our approaches to living with it. The results reveal three key dynamics: the negotiation of algorithmic authority, the limitations of measuring and categorising sleepwalking and the importance of integrating algorithmic data with personal experience. Our findings show that algorithmic systems do not simply detect sleepwalking; rather, the relationship between sociotechnical processes, clinical routines and knowledge, self‐tracking practices and user interpretations constitutes it. Thus, sleepwalking can only be understood through the negotiation of data, narratives and embodied experiences, which reveals the limitations of standardised algorithmic measurement and the necessity of integrating diverse epistemic resources. These findings make a valuable contribution to sociological theory by shedding light on the relationship between human action and algorithmic influence in healthcare.

## 1 Introduction

Sleepwalking or somnambulism is a phenomenon that blurs the boundaries between wakefulness and sleep. It is characterised by complexity and unpredictability: Sleepwalkers have no direct influence on their behaviour and are largely amnestic for it. Because of this indeterminacy, sleepwalking poses a challenge not only in everyday life but also in a medical context, as it cannot be easily and unambiguously identified. Such uncertainties are often countered using digital technologies and algorithmic systems to identify, categorise and treat health conditions (Amelang and Bauer 2019; Ruckenstein and Schüll 2017; Schwennesen 2019; Zheng et al. 2024; Zillien 2020). These technologies play a crucial role in identifying and monitoring the underlying causes and patterns of sleep disorders in both medical and private contexts. Sleep medicine facilities and digital self‐tracking apps use technological devices to measure, for example, heart rate and movement. The collected data are then evaluated using algorithmic models to identify and classify sleep patterns according to sleep medicine criteria. Consequently, the intertwining of science, sleep and technology can nowadays no longer be seen as a sociophysical process but instead more as a form of ‘technosleep’ (Coveney et al. 2023), in which sleep is primarily understood as an object of technological measurement and calculation.

This tendency towards technologisation also shapes the relation to sleepwalking. Classified as a neurological and behavioural disorder, it is typically diagnosed through video‐assisted polysomnography (PSG). This involves recording neurological, muscular and respiratory activity, measured via electrodes and sensors, combined with audio‐video monitoring during sleep and patients' descriptions of suspected sleepwalking episodes (Reinhardt 2023). Although sleep disorders such as insomnia are comparatively well definable, identifiable and quantifiable, sleepwalking—as a parasomnia—can manifest in ambiguous ways and remains difficult for automated systems to detect (Idir et al. 2022; Picard‐Deland et al. 2025). Either the technological systems are not suitable for direct detection, or movement artefacts complicate recognition. Furthermore, patients' behaviour is affected by the medical environment (Lopez et al. 2023; Picard‐Deland et al. 2025). A similar situation arises in the private sphere: Digital self‐tracking technologies similarly rely on bodily measurements and algorithmic processing to construct sleep patterns and highlight deviations (Lyall 2021; Nansen et al. 2021; Wettmann 2026; Williams et al. 2015; Zillien et al. 2023). However, these devices do not directly measure sleepwalking. Instead, they calculate sleep based on physiological data and visualise them in graphs and figures, from which sleepwalking occurrences can then be deduced retrospectively by the users.

Algorithmic processing of physiological data is key to identifying, categorising and visualising sleep disturbances by comparing them to typical sleep patterns. However, these systems struggle with sleepwalking because they are not designed to identify it. Sleepwalking remains hard to detect and indistinguishable from other sleep patterns because it does not produce measurable physiological signals but instead manifests as behaviours that require external interpretation. Nevertheless, algorithmic tools remain widely used, driven by the prevailing logic of quantifying sleep. In our analysis, we therefore focus explicitly on the role of algorithmic systems in the detection and treatment of sleepwalking from a relational perspective (Dourish 2016; Seyfert 2022, 2024), highlighting the epistemic, social and infrastructural limitations of algorithmic systems in a clinical context and in the use of sleep‐tracking technologies. Based on a multisited ethnographic dataset collected between 2020 and 2025, we combine observations in sleep medical facilities with blogs and forums about sleep tracking, sleepwalking videos on social media platforms and interviews with sleepwalkers. Together, these materials provide a comprehensive view of both the clinical and the everyday technological dimensions of sleep and sleepwalking. Based on this, we examine how algorithms highlight measurable data, shape relationships and construct phenomena. In doing so, we argue that sleepwalking, in both the medical and private spheres, is formed through the negotiation of different perspectives and thus as a synthesis. Therefore, sleepwalking is constituted by its sociotechnical negotiation. We show that sleepwalking emerges through situated practices of algorithmic mapping, interpretation and negotiation across clinical and everyday contexts.

In the following, we first outline the conceptualisation of sleepwalking as a medical problem and our sociological understanding of algorithms. Then, we provide a detailed overview of our methodological approach. We then discuss our empirical analysis on the mapping of sleepwalking in sleep medicine facilities and via sleep tracking. Our analysis highlights three key themes: (1) the use of algorithms to gain insight into sleepwalking; (2) the limitations and tensions involved in measuring and categorising sleepwalking; and (3) the importance of combining different perspectives, such as algorithmic data with narratives and embodied experiences. Finally, we conclude with a summary of our key findings.

## 2 Towards the Problematisation of Sleepwalking

Despite its widespread occurrence,^1^ sleepwalking as a threshold phenomenon remains largely unexplored. This is partly because it often has only a minor direct impact and thus often receives limited attention or goes unnoticed. Sleepwalking often becomes apparent only when problematic situations arise, third parties point it out, or visible signs of physical irritation or disturbance occur, creating challenges for sleepwalkers and those around them (Reinhardt 2023; Wettmann 2025a). In everyday life, sleepwalking is thus problematised primarily when it produces noticeable consequences in a social environment. At the same time, sleepwalking manifests in varying degrees of passive corporeality (Wettmann 2025b), ranging from speaking or moving in bed to sitting up, walking around, performing physical labour and, in extreme cases, self‐harm or aggression towards others.^2^ This broad behavioural spectrum makes it challenging to recognise sleepwalking as a coherent phenomenon, as many manifestations are ambiguous or only interpreted retrospectively.

Medically, the International Classification of Diseases (ICD‐11) classifies sleepwalking as a ‘parasomnia disorder’ under the heading ‘disorders of arousal from non‐REM sleep’, which refers to ‘problematic behavioural or physiological events’ and defines somnambulism as a disorder that ‘is characterised by ambulation and other complex behaviours during a partial arousal from deep sleep’ (WHO 2019). The current Diagnostic and Statistical Manual of Mental Disorders (DSM‐5), primarily used in the United States, also recognises sleepwalking as a problematic phenomenon that can be classified and defined as follows: ‘Repeated episodes of rising from bed during sleep and walking about. While sleepwalking, the individual has a blank, staring face; is relatively unresponsive to the efforts of others to communicate with him or her; and can be awakened only with great difficulty’ (APA 2013). Additionally, the DSM‐5 mentions exhaustion and fatigue as impacts on the sleepwalker's daily life, as well as possibly dangerous occurrences for the sleepwalker or other people. All these classificatory systems emphasise potential risks, fatigue and daily impairments. In clinical practice, other phenomena are scaled in the context of sleepwalking; for example, somniloquy (talking or shouting during sleep) and sexsomnia (performing sexual acts during sleep). Despite not having a specific ICD‐11 code and mostly being subsumed under sleepwalking events, these are sometimes considered to be on the same spectrum as sleepwalking, or, at least, closely related to it (Heidbreder 2020b; Peter 2020).

In this sense, sleepwalking is not merely a nocturnal event but a product of diagnostic practice. Like other parasomnias, it emerges where behaviour is classified, bodies are recorded, deviations are named, and phenomena are ordered (Zifonun et al. 2023). The indeterminacy of the sleepwalking state is thus translated into a category within a system that seeks to diagnose, treat and regulate. However, sleepwalking is challenging to diagnose, as it needs to occur in a medical setting first and then be ruled out from other disorders with similar symptoms. In sleep medical facilities, sleepwalking is induced using triggers such as alcohol consumption, forced sleep interruptions, stress, sleep deprivation, certain medications or the presence of an intimate partner (Arnulf 2018; Zadra et al. 2013). However, sleepwalkers' amnesia and vague self‐descriptions are often insufficient for differential diagnosis. Even video recordings brought by patients that document—at least in their interpretation—sleepwalking behaviour usually require joint interpretation by clinicians and patients to express a preliminary suspicion. Sleepwalking is not immediately recognisable, even in PSG recordings, as the transition between sleep phases is a regular part of the sleep process. Making a diagnosis of sleepwalking is therefore anything but easy.

## 3 The Sociology of Knowledge on Algorithmic Mapping

Algorithms classify, filter, evaluate and prioritise data in fields ranging from financial markets, social media, medical practice and public administration (e.g., Beer 2017; Burrell and Fourcade 2021; Donia 2025; Henwood and Marent 2019; Langenohl 2021; Ziewitz 2016). Their outputs not only structure technical processes but also intervene in social orders and help determine essential processes. Algorithms are therefore anything but neutral. The development of algorithms requires training databases already embedded in complex social contexts (Marres 2017, 233). They are not merely objective tools but also carriers of social rationalities, shaped by specific ideas and interpretative patterns. Therefore, the debate on algorithms should not be based purely on the technological aspect but also on the situations in which they are developed, used and have an impact: Algorithms ‘need to be analyzed and understood within those systems of relation that give them meaning and animate them’ (Dourish 2016, 2). This highlights the importance of a *relational understanding of algorithms*. Their meaning does not derive from an internal technical logic but from their relational embedding in practices, data structures, programmes and institutional contexts.^3^ Thus, the way in which algorithms exert their influence and gain validity is the result of cultural meaning and knowledge production, institutional classification processes, their material implementation and specific infrastructures (Dourish 2016).

We conceptualise these relational effects as *algorithmic mapping*: sociotechnical processes that organise, compare and operationalise realities. From this perspective, Seyfert (2022) examines algorithms in relation to the impact of regulatory mechanisms surrounding them. He points out that regulation is not to be seen simply as a response to algorithmic technologies but as a formative agency that participates directly in their construction (Seyfert 2022, 1542). Algorithms are therefore not constituted neutrally; rather, they are shaped by normative assumptions about market behaviour, subjectivity and transparency. Beyond institutional conditions, Seyfert (2024) examines the relational dynamics between humans and algorithms, defining ‘algorithmic sociality’ as a perspective that rejects the dichotomy of humans as primary actors and algorithms as dominant structures. Instead, relational entanglements are to be considered, in which algorithmic sociality can unfold in the first place. Drawing on Simondon's concept of disparity—a synthesis that preserves differences between dissimilar elements rather than resolving them (Seyfert 2024, 37)—Seyfert argues that humans and algorithms form interdependent relationships characterised by a ‘metastable equilibrium’ (Seyfert 2024, 26, translated). This process is one of continuous interrelation and co‐production.

Rather than taking an instrumental or technology‐deterministic approach to the ‘social power of algorithms’ (Beer 2017), Seyfert starts from a relational conception that seeks to understand them in terms of their relationships. He argues that the use of algorithms is shaped by ‘vernacular knowledge’ (Seyfert 2024, 39, translated), defined as everyday, practical knowledge developed through interaction. This generates ‘performative effects of *digital practices*’ (Seyfert 2024, 40, translated, emphasis in original), evident in the continuity of algorithmic use and its consequences. Algorithmic sociality is thus understood as a process in which meaning, behaviour and structure interact with one another. The relationship between humans and algorithms is conceived here as a place of co‐production of perception, knowledge and action and thus as a social formation in which technical and human elements are inextricably linked. At the same time, a wide variety of algorithms interact with one another, which is why attention must be paid not only to the relationships between digital environments, technological systems and actors but also to ‘inter‐algorithmic relationships’ (Seyfert 2024, 35, translated).

Such an understanding of algorithms not only describes new forms of relationships but also opens the possibility of reconsidering the epistemic conditions of digital knowledge production. Sleepwalking, as a phenomenon of physiological and cognitive ambiguity, offers a paradigmatic case for such epistemic reconsideration. It exposes how algorithmic procedures—initially developed for stable, quantifiable forms of sleep—encounter the unstable, drifting and ambiguous behaviour of sleepwalkers, forcing a reconfiguration of what can be known and classified at all. Algorithms do not simply produce knowledge but actively contribute to the creation of what is considered relevant, visible or diagnosable. This knowledge is situated, contingent and relational, emerging from interactions between data, practices, expectations and sociotechnical rules. We therefore analyse algorithmic systems sociologically as *mapping processes*. Algorithms do more than just map reality; they actively cocreate it by organising specific data, linking them to defined categories and thus making deviations visible while obscuring other aspects. This mapping process is particularly evident in sleepwalking because it cannot be calculated directly. Instead, it is derived and synthesised from a combination of various situational elements and data. Sociotechnical negotiation, rather than mere technological recording, is demanded by sleepwalking, as we demonstrate in the following analysis.

## 4 Methods

This study is part of the research project ‘Sleepwalking: Recalcitrant Knowledge about a Liminal State’ (project number 436267559), funded by the German Research Foundation. The first sociological project group examines the professional environment of sleep medicine and research, whereas the second analyses the use of digital technologies in private contexts. In line with grounded theory (Glaser and Strauss 2010; Strauss 1987), data from both groups were merged and evaluated together to reconstruct the production of knowledge about sleepwalking in algorithmically influenced societies. The first group examines the practices of scientific knowledge generation in *sleep medical and research facilities* (Svenja Reinhardt). Using ethnographic methods, the epistemic orders and institutional logics that structure the medical classification and interpretation of sleep medicine and sleepwalking were reconstructed. The data include (lifeworld‐)ethnographic (Honer and Hitzler 2015; Breidenstein et al. 2020) material from fieldwork conducted in mainly two German inpatient and outpatient sleep medical facilities (plus eight more for short‐term stays) from 2021 to 2025 and two European sleep research facilities, including participant observation of sleep medical practices such as patient preparation, data evaluation, discussion of findings, diagnosis and therapy, as well as 24 field conversations and interviews with practitioners, case studies of 10 patients, 11 interviews and several hundred field contacts with patients during the 62 nights and days of fieldwork in the sleep medical and research facilities and beyond. Additionally, 46 short‐term and 15 long‐term workshops, training sessions and talks in the realm of sleep research and medicine were observed.

The second group focuses on *digital sleep‐tracking* *practices and digital representations of sleepwalking* (Nico Wettmann). Here, the empirical analyses are based on self‐reports, posts on discussion forums and video recordings from community conferences and social media platforms such as Instagram and TikTok. Thus, practices, discourses and representations of sleep and sleepwalking by laypeople were examined using an online ethnographic approach (Hine 2008, 2015). Ultimately, 25 blog posts, 62 Reddit threads from various subreddits on sleep, sleep tracking or biohacking and 29 videos on sleep tracking were exported and transcribed for further analysis. These included nine videos of quantified self‐meetings from 2011 to 2016, 10 video reviews and comparisons of sleep‐tracking technologies and 10 recordings of the Biohacker Summit from 2016 to 2022. Additionally, 40 threads from different online forums on sleepwalking were identified and integrated, as well as 80 Reddit threads from various subreddits and 60 TikTok, Instagram and YouTube videos showing sleepwalking behaviour. The empirical material was collected through ‘lurking’ (Hine 2008, 262) in online communities and through social media posts found using the search function with various search terms such as ‘sleep’, ‘sleepwalking’ and ‘sleep tracking’ and by following the links between websites and posts. Thereby, only public, freely accessible online forums, blogs and subreddits, as well as the websites of the quantified self and biohacking communities and manufacturers of self‐tracking devices, were considered for this online ethnography. In terms of research ethics, the accessibility and sensitivity of the available online material were considered during data collection (Heise and Schmidt 2014, 528). This meant that only publicly accessible material, findable via search engines without registration or passwords, was included (Döring 2013, 311). Following the principle of ‘theoretical sampling’ (Glaser and Strauss 2010, 61, translated), information on sleep and sleepwalking in relation to digital technologies was sought from relevant posts and videos on online forums, websites and social media. Content dealing with sleep, sleepwalking and the production of knowledge on these topics, which problematised them, was then included in the data collection. In addition to these materials, both authors jointly conducted 20 interviews focusing on sleepwalking: nine with sleepwalkers and 11 with experts. This was done to gain insights into their experiences with the recognition and diagnosis of sleepwalking and to learn how it is dealt with in medical and everyday contexts.

Juxtaposing these two sociological research approaches enables a systematic reconstruction of the tensions between knowledge bases, digital self‐observation and the cultural appropriation of sleepwalking as a liminal phenomenon. We analysed the manifest empirical materials (ethnographic observations, interviews and online interactions) to reconstruct the latent processes through which meaning, agency and algorithmic interpretation emerge. From these materials, we inductively identified latent analytical objects, namely, doctor–patient interaction, algorithmic decision‐making and user negotiation of self‐tracking data. These are not direct objects of observation but reconstructed interpretative structures that organise the manifest practices. Within this framework, agency refers to these latent dynamics—to how humans and algorithms co‐produce interpretative space, respond to uncertainty and redefine epistemic authority within sociotechnical environments. Although our empirical focus lies on observable practices and data sources (interviews, field observations and online interactions), our analytical lens addresses the latent structures that underlie these practices—particularly how agency manifests in the negotiation between human interpretation and algorithmic mediation. After individual analyses, we triangulated findings on algorithmic systems in sleepwalking through shared data sessions and memo discussions. This iterative process—comparing and calibrating insights—refined our analytical categories and highlighted thematic intersections and frictions informing the subsequent discussion.

## 5 Mapping Sleepwalking in Sleep Medical Facilities and via Sleep Tracking

In the following, we examine the algorithmic mapping of sleepwalking in medical facilities and digital self‐tracking. We focus, first, on how sleepwalking is deduced and negotiated through the measurement of sleep using media technologies employed in these areas; second, we address the challenges of algorithmic mapping that arise in both contexts and how the respective actors respond to them; and third, we argue that an integration of perspectives occurs in both settings, as algorithmic mapping always constitutes only one component in the construction of sleepwalking as a phenomenon.

### 5.1 Algorithmic Negotiation of Sleepwalking Through Sleep Mapping

A foundational concept in the context of sleep medicine and digital self‐tracking regarding sleepwalking is the algorithmic production of sleep itself, described as ‘m‐apping’ (Williams et al. 2015, 1050) or the ‘fabrication of sleep’ (Zifonun 2025). These concepts do not refer to neutral measurement but to a relational process of knowledge production, in which heterogeneous physiological signals are translated into standardised categories of established sleep medical models. Through this process, sleep is constructed as an ordered sequence of stages that serves as a normative framework. Only against this background can deviations be identified and interpreted. Sleepwalking, understood as a sleep disturbance, thus does not appear as a directly measurable phenomenon but becomes intelligible only in relation to what the device counts as ‘standard sleep’ (Lyall 2021, 149). Algorithmically, the detection of sleepwalking is therefore conditioned by the prior production and stabilisation of sleep itself.

In the sleep medicine facilities we observed, this is evident in the measurement of patients' sleep and the evaluation of several PSG recordings based on algorithmic procedures.^4^ A typical protocol spans three nights: On the first night, a baseline PSG is conducted to measure initial data and capture any spontaneous sleepwalking. On the second night, a suspected trigger such as alcohol or sleep deprivation is introduced. On the following night, another PSG is performed to detect signs of sleepwalking triggered the night before. Algorithmic analyses refer here primarily to automated signal processing, segmentation and rule‐based classification procedures that prestructure the PSG data before clinical interpretation takes place. Raw physiological signals recorded via, inter alia, electroencephalogram (EEG), electrooculography (EOG) and respiratory sensors are first digitally filtered and segmented into standardised time epochs of typically 30 s. Within these epochs, trained algorithmic scoring routines are used to mark characteristic signal patterns, such as frequency bands in the EEG, specific eye movement activity in the EOG, muscle tone and movement variations in the electromyography (EMG), or breathing irregularities derived from airflow and thoracic sensors to identify hypopnoeas or apnoeas. Based on these pattern recognitions, sleep stages are algorithmically categorised as wake, N1 (falling asleep), N2 (light sleep), N3 (deep/slow‐wave sleep) and REM (rapid eye movement sleep). These classifications rely predominantly on rule‐based algorithms derived from sleep medical knowledge rather than on autonomous machine learning models. The formal criteria for this classification are defined by the American Academy of Sleep Medicine Manual (AASM 2023), which specifies threshold values, signal combinations and decision rules for sleep staging and event detection (e.g., apnoea, hypopnoea, arousals or periodic limb movement). Importantly, these algorithmic procedures do not, in themselves, produce a diagnosis for legal purposes. Rather, they generate a structured representation of sleep that highlights potential deviations and events. Trained sleep technicians and physicians subsequently review, correct and contextualise these algorithmic outputs, for instance, by rescoring ambiguous epochs, interpreting video recordings or relating detected events to patient narratives and situational factors. Algorithmic analysis thus functions as an infrastructural layer that organises and prioritises the data, whereas diagnostic meaning emerges only through its integration into clinical interpretative practices.

However, the detection and confirmation of sleepwalking depend on how behavioural anomalies are calculated algorithmically to translate fluid physical states into discrete, measurable stages. Most sleepwalking episodes occur in the first half of the night, when deep sleep is predominant. In these cases, EEG readings may show a paradoxical mixture of wake‐like activity and deep sleep patterns accompanied by parasympathetic arousal; these are sometimes associated with parasomnias in clinical interpretation. Additionally, movement patterns characterised by irregular EMG activity, video‐assisted interpretation and specific EEG patterns are examined, as these may signify sleepwalking episodes. Arousals are noted to differentiate parasomnias from other forms of nocturnal activity. The evaluation of sleep in clinical settings through PSG yields extensive data, yet the presence of sleepwalking must be manually interpreted. This interrelationship between automated classification and human expertise can be described as ‘algorithmic assemblages of care’ (Schwennesen 2019). This is particularly evident in clinical practice, where algorithmic results are processed and selectively integrated into clinical routines:

Once an evaluation has been completed, the computer calculates a few things, which [medical‐technical assistant (MTA)] then prints out. The printouts contain several graphs as well as a list of various data points computed by the PC. The AHI [apnoea‐hypopnoea index per hour of sleep] and sleep efficiency seem to be particularly important, as [MTA] copies these values by hand into the patient record on the routing slip. He files the printed evaluation sheets in another section of the patient’s chart.

(Translated)

Diagnostic processes in sleep medical facilities therefore involve not only technical measurements but also interpretative selection. Metrics such as the apnoea‐hypopnoea index (AHI) and sleep efficiency are prioritised, whereas the relevance of other data depends on the context. This ‘copying by hand’ is not to be disregarded as trivial, as it marks a point where algorithmic calculation becomes socially valid ‘objective’ medical knowledge through human mediation. Because most PSG systems prioritise sleep‐related breathing disorder (SRBD) detection, parasomnias such as sleepwalking require additional interpretative and evidentiary steps. Here, the seemingly mundane description of ‘the computer calculating a few things’ exemplifies how algorithmic prestructuring shapes what becomes diagnostically relevant, as the MTA does not simply transfer neutral data but engages in selective translation—guided by the algorithmic output and institutional expectations. The algorithmic systems thus focus on SRBD, indices and movement disorders, overlooking many parasomnias such as sleepwalking.

**FIGURE 1:**
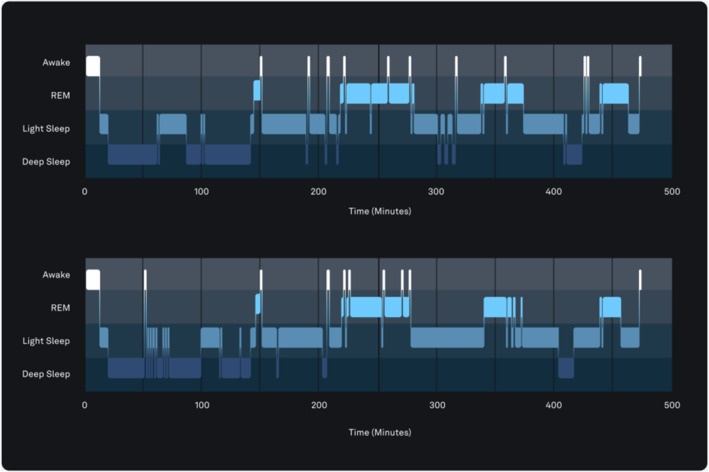
Comparison of hypnograms measured with PSG (top) and Oura (bottom), with 84% of the measurements matching. *Source:* Oura (2022).

Digital self‐tracking technologies similarly attempt to make sleep measurable through quantification and classification (Lyall 2021; Nansen et al. 2021; Wettmann 2026; Williams et al. 2015), with some users also attempting to register somnambulistic episodes. This is carried out using a range of sensors—including accelerometers, temperature sensors and infrared photoplethysmography—through which common self‐tracking technologies collect physiological data and process them through sleep‐stage classification algorithms. In their analysis and interpretation of sleep, these self‐tracking technologies align closely with the ‘standardised packages’ (Fujimura 1992) of the sleep medical facility, that is, the established epistemic formats and knowledge bases of clinical practice. For example, Oura's algorithm, one of the most widely used self‐tracking technologies in the field, approximates sleep based on acceleration, pulse, heart rate variability and temperature measurements, combined with knowledge from the field of sleep medicine (Altini and Kinnunen 2021; Svensson et al. 2024). During its development and ongoing adaptation, the Oura algorithm was repeatedly compared with PSG‐based sleep staging and adjusted accordingly. These iterative comparisons with PSG data exemplify a form of algorithmic calibration in which clinical standards become embedded into consumer devices, thereby extending institutional epistemologies into everyday life. Ultimately, a hypnogram is a visual representation of a person's sleep pattern over the course of a night, showing the different sleep phases and their durations. Here, the algorithm classifies sleep over time into four stages: awake, REM, light sleep and deep sleep. Therefore, the development of sleep trackers based on algorithms consistently draws on a reduced, more colloquial version of valid knowledge within sleep medicine, thereby legitimising itself (Figure 1).

Based on medical ‘normal sleep’ and the calibration of self‐tracking technology algorithms to the gold standard in sleep medicine, video‐assisted PSG, widespread sleep disorders such as sleepwalking remain insufficiently defined and are not truly measurable. Users of digital self‐tracking systems therefore rely on their own ‘data sense‐making’ (Lupton 2017, 1604) of their sleep data to detect sleepwalking. An interviewee describes how she interprets the self‐tracking data on her device as indicating sleepwalking as follows:

I’m wearing the watch here [points on watch] (…) and it shows me my sleep stages and how long I’ve slept. (…) Yes, that’s when it registers, it’s displayed as a waking state. It’s a thin line, on my watch it’s a thin pink stripe that appears. It indicates the waking state. That actually confirmed once again that this is the case in the first 2 to 3 hours.

(Translated)

Through interpretations of existing data, as in this case, sleepwalking can only be deduced from sleep records if self‐trackers are aware of their sleepwalking tendencies and understand which data values and graphs can be used to identify them. Outliers and deviations from expected sleep data can then be interpreted by users as sleepwalking, especially because the algorithms of self‐tracking technologies often incorrectly interpret somnambulistic movements as lighter sleep phases, brief awakenings or bodily movements during sleep, as it is stated in the interview. Therefore, sleepwalking remains largely invisible as these systems favour regular, continuous patterns over irregular, socially conditioned events. This omission illustrates that sleepwalking is not only overlooked by algorithms but also excluded by the models on which they are based.

Our analyses show that, in both contexts, sleepwalking is not only observed but also actively mediated and co‐constituted by digital technologies and their algorithmic infrastructures. These algorithms are designed to recognise ‘normal’ sleep as a sequence of standardised phases and detect isolated disturbances only if they correspond to predefined parameters. However, complex phenomena such as sleepwalking evade these conditions and expose the sociotechnical vulnerability of algorithmic epistemology. This fragility highlights the need for a relational understanding of digital knowledge production that goes beyond automation.

### 5.2 Challenges of Algorithmic Mapping

Although algorithmic mapping enables the constitution of sleepwalking as an object of knowledge, it simultaneously produces systematic limitations and tensions. Rather than being directly detected, sleepwalking remains a fragile achievement that depends on contextual signals and interpretative labour in both clinical and self‐tracking contexts. In the following analysis, we therefore examine where and how algorithmic mapping reaches its limits. Although algorithmic systems promise objectivity, consistency and efficiency, detecting sleepwalking remains a fragile achievement in both clinical and self‐tracking contexts. Rather than being directly identified, sleepwalking is inferred through contextual signals and interpretative labour. In clinical settings, automated scoring is routinely adjusted by technicians, highlighting the provisional and negotiated character of algorithmic authority. Crucial cues—such as EEG deviations in N3, microarousals, video‐based interpretations or EMG activity—may suggest sleepwalking but can just as easily indicate other conditions or nondefinitively classified movements. The relevance of these cues depends not only on their occurrence but also on their contextualisation within the patient's profile. In the context of an AI‐based scoring project, one technician commented: ‘We still need a human to look over it’ (translated). This statement encapsulates the relational character of algorithmic knowledge and its dependence on interpretative, embodied work (Schwennesen 2019): Algorithmic systems thus do not replace expertise but redistribute it, creating new forms in which professionals mediate between computation and lived experience. A central limitation lies in standardised training datasets, as they are mostly optimised for SRBDs or otherwise healthy patients. As a result, algorithms may flag typical patterns as deviations or overlook diagnostically relevant irregularities. Because SRBDs are more frequent, easier to detect and more treatable, they dominate algorithmic and clinical attention, leaving sleepwalking largely marginalised within automated systems.

In everyday life, self‐tracking technologies are often used to address uncertainties and provide clarity about one's situation (Zillien 2020). However, as previously mentioned, the apps do not consistently recognise or interpret sleepwalking based on medically recognised measurements. Users therefore identify such events themselves by observing deviations from their usual sleep patterns and tracking the irritations they experience as a result of the data. In an online forum, one user sought advice after noticing strange patterns in their data.

Last night I had the weirdest result. I went to sleep at midnight, but it showed that my iPhone was moving until 2AM and my heart rate peaked at 140 around 1:30AM. My girlfriend said she did not move my phone and I’ve never sleepwalked in my life (nor did she notice me getting up). (…) Anyone else had this kind of issue? I’m doubting if it’s an error on their side or if I’m doing crazy things at night.

On reviewing the data, the user notices movement patterns and an increased heart rate during the night. As he does not remember getting up, he wonders whether this is a technical error or sleepwalking. The interpretation of the deviation from previous sleep measurements sparked uncertainty and interest in what happened that night and whether it could have been sleepwalking. In the forum, others respond that sleepwalking is the most plausible explanation given the physiological activity. Access to sleepwalking is thus not achieved through direct detection using self‐tracking technologies. Rather, what occurs during measurement remains largely indeterminate. At the same time, sleepwalking is not conceptualised as a disorder because it is assumed to be a universal phenomenon. Any deviation from this norm and expected pattern can therefore be interpreted as sleepwalking, given that sleep itself is understood as a fundamentally passive process. Yet, uncertainty persists as to whether such deviations or nocturnal movements reflect actual phenomena or are merely artefacts.

On the contrary, one sleepwalker documented an experiment on his blog, trying various self‐tracking devices to capture his episodes. Yet he found none of them reliable enough, ultimately concluding: ‘No sleep tracker detects my sleepwalking’. This reflects a general pattern among the sleepwalkers we interviewed: When sleep data are collected via self‐tracking, they rarely inform cases of sleepwalking. As a result, some users attempt to bridge this gap through vernacular experimentation (Neff and Nafus 2016), manually comparing movement peaks, heart rate fluctuations and reported awakenings to infer possible episodes—turning algorithmic feedback into an interpretative practice. In doing so, they transform data analysis into an everyday practice, reclaiming agency within systems that were not designed to recognise their experiences. For example, one interviewee told us that the tracking data on their own sleepwalking were not very useful but rather served as general information:

I also tracked my sleep with the Apple Watch. But well, I couldn’t really see much—just how much deep sleep I had, and so on. I saw I had taken a few steps now and then (laughs). […] It was more informative.

(Translated)

Across both settings, the mapping of sleepwalking exposes fundamental tensions between automation and ambiguity, standardisation and individuality, data and experience. As sleep trackers seek to gain insight into their own sleep, a further limitation emerges: They remain asleep during the very episodes they aim to observe, and they must rely entirely on technological approaches, whereas in clinical settings, trained personnel interpret the occurrences. Ultimately, sleepwalking becomes knowable only through practices of negotiation, bricolage and meaning‐making—rather than as a stable metric or fixed signal. These limitations do not render sleepwalking unknowable. Instead, they necessitate the integration of heterogeneous perspectives through which sleepwalking is made intelligible in practice.

### 5.3 Integration of Perspectives

Against this backdrop, we argue that sleepwalking, as a complex phenomenon, precludes the development of a simple diagnostic algorithm. Sleepwalking cannot be objectified in this way. This is particularly evident in algorithmic mapping, where the reduction of contingency through sociotechnical measurements of sleepwalking is limited. Unlike other sleep disorders, subjective narratives are not merely supplementary sources of knowledge; they are a necessary component of understanding sleepwalking, both medically and in everyday contexts. In a pragmatic sense, diagnosing and determining sleepwalking involve a forensic search for clues resulting from the integration of various ‘practical judgements’ (Dewey 1915, 505). Therefore, it is insufficient to interpret sleepwalking solely using PSG and self‐tracking technologies; various perspectives must also be considered, such as morning experiences or third‐party descriptions. By integrating perspectives, sleepwalking can be rendered practically intelligible through the coordination of heterogeneous forms of knowledge.

In the medical field, video‐assisted PSG, where multiple data streams converge, presents a technical and interpretative challenge. Clinicians must navigate between algorithmic results, embodied observations and institutional constraints to make sleepwalking visible and treatable. Although PSG provides a detailed physiological representation of sleep phases, movements and states of arousal, it rarely focuses on parasomnias. This tension is illustrated by the frustration of one patient after her sleepwalking diagnosis; she recalls the diagnostic and therapeutic process:

It’s frustrating, when you have to go into the hospital for days just so they can tell you what you already know—and then they give you medication that doesn’t work and makes you feel worse.

(Translated)

Other forms of treatment besides a sedative, such as autosuggestive training or CBT‐I, were not presented to her as possible options. When she informed her doctor that the treatment was ineffective, the dosage was simply increased. Her account reveals disappointment in the therapy and highlights the disconnection between the technical diagnosis and her lived experience. Here, the highly standardised clinical logic led to diagnostic closure without therapeutic resolution, as clinical classifications can stabilise formal knowledge without improving care. Such reports reveal how fragile the connection among data, interpretation and practical help can be. It also becomes clear that integration is not only an epistemic process but also an institutional and emotional condition that requires time, responsiveness and a willingness to deviate from protocols. Integration, in this sense, results from a combination of data accumulation and the active orchestration of perspectives against systemic inertia.

However, in self‐tracking contexts, knowledge about sleepwalking can only be obtained by considering different viewpoints. Rather than relying on quantified values, those affected tend to use audio and video recordings to reconstruct what happened. In an online forum, for example, it is emphasised that sleepwalking can only be recognised through such recordings for it to be problematised and for somnambulistic episodes to become approachable. Thus, sleepwalking is not just about the quantitative assessment but specifically about the quality of sleepwalking. In contrast to self‐tracking, one user emphasises the necessity of video recordings: ‘I have to SEE what is going on’. In this sense, one of our interviewees uses audio recordings to reconstruct what happened while sleepwalking. First, she looks at her self‐tracking data for periods of wakefulness and movement. Then, she evaluates the corresponding audio recording and listens to the identified sections. This enables her to determine, in her opinion, whether she experienced a sleepwalking episode and what happened during it:

I just listened to what I had been doing during the night. […] I marked a few that were kind of interesting. I laughed about them briefly or something [laughing]. What else could I do? […] So it was mainly for me, but also for the sleep doctors. Back when I introduced myself to both doctors, I also played them a few recordings.

(Translated)

She uses her records to gain insight into her sleepwalking and determine what happened during the night. This makes her sleepwalking behaviour more accessible and sometimes enables her to understand why it occurred. However, for sleepwalkers themselves, this information is often merely interesting. Although they know what was said and done, nothing else is done with this information except, perhaps, to amuse themselves. On the other hand, such records can serve as evidence in a medical context, facilitating access to a sleep medical facility and a medical diagnosis. In an interview with us, a sleep medicine doctor emphasised the value of patient records.

That can be very helpful. So, good video material from patients is extremely helpful. And I understand, as I said, because most episodes do not happen in the sleep laboratory.

(Translated)

Apart from measuring sleepwalking through technological quantification and visualisation, audio and video recordings can also help individuals recognise their own sleepwalking and understand what happens during these episodes. Technological data must then be linked to these recordings to identify possible sleepwalking. In addition to these records, reports from third parties who witnessed these incidents are particularly important. Our analysed sleepwalkers repeatedly emphasise that they only found out about their condition from their parents, partners or family members, as recalled in an interview: ‘I heard stories from my mum about how I used to walk around or tell stories’ (translated). However, due to the lack of direct experience, assessments of sleepwalking are essentially based on accounts from family members and other third parties who have witnessed the somnambulistic situation. Knowledge about one's own sleepwalking is therefore derived from secondary interpretations and accounts. This offers an interpretation of one's sleepwalking, making it a biographical normality.

Knowledge about sleepwalking is expanded by integrating various reports, traces and records that make one's own behaviour during an episode tangible. Our findings demonstrate that sleepwalking cannot be adequately reconstructed as a purely biomedical or data‐based phenomenon. Rather, different perspectives are integrated and coordinated: reports from third parties who have observed sleepwalking, the interpretation of local traces in the room and on the body that indicate unconscious behaviour, and media technology recordings that visualise sleepwalking activities. Through the coordination of these heterogeneous perspectives, sleepwalking becomes intelligible in practice. Integration, in this sense, does not eliminate uncertainty but instead renders sleepwalking tangible by linking medical classifications, technological records and everyday interpretations through the integration of different sources of knowledge. Not only because algorithmic systems cannot calculate sleepwalking but also because the phenomenon, as a ‘boundary object’ (Star and Griesemer 1989), constitutes itself and becomes tangible only through the integration of medical classifications and everyday irritations through sociotechnical practices of mapping and interpretation. Sleepwalking thus emerges from the situated integration of medical classifications, technological traces and everyday interpretations. It is produced neither by algorithms nor by diagnosis alone but through the ongoing negotiation between medical classifications, technological traces and lived experience.

## 6 Concluding Discussion

Sleepwalking exposes the limits of algorithmic rationality by confronting digital systems with an event that resists—at least for now—measurement and prediction. It disrupts the logic of automation that privileges regularity and calculability, demonstrating that knowledge in algorithmic environments is always relational, situational, negotiated and contingent. In the clinical context, video‐assisted PSG demonstrates that algorithmic detection alone is insufficient: Although it provides detailed physiological data, sleepwalking is interpreted in the context of other evidence, such as patient narratives or video recordings. In the context of self‐tracking, these negotiations shift from the institutional to the individual level, where individuals give meaning to ambiguous graphics and movement data through everyday practices and in connection with their own body awareness (Lupton 2017; Pantzar and Ruckenstein 2017). Thus, the two settings mirror one another as distributed forms of agency: institutional calibration on one side and personal experimentation on the other.

In contrast to previous research on digital sleep and self‐tracking, sleepwalking exposes the limits of what can be algorithmically sensed or known. It foregrounds tensions within algorithmic mapping between standardisation and ambiguity, automation and experience, visibility and invisibility. As a phenomenon that is both embodied and imperceptible to the self, sleepwalking marks the point where datafication fails to capture lived experience. In tracing how it resists classification, this article demonstrates that the negotiation of meaning in algorithm‐based healthcare is not peripheral but central to how knowledge and agency are distributed. Sleepwalking thus extends debates on ‘technosleep’ (Coveney et al. 2023) by showing that ambiguous and embodied events do not remain unrecognised by algorithmic systems but are actively shaped through their classificatory logics. These systems structure what becomes visible and actionable yet remain dependent—in both presented fields—on human interpretation. The resulting interplay reveals two interrelated forms of agency: (1) algorithmic agency, which organises attention and relevance, and (2) interpretative human agency, which recontextualises and negotiates these outputs. In both clinical and everyday contexts, sleepwalking is co‐produced through this recursive relation between algorithmic structuring and interpretative labour. As we have shown in our analyses, sleepwalking becomes intelligible not through algorithmic detection alone but through the integration of heterogeneous perspectives across clinical routines, technological infrastructures and everyday practices.

Across both domains, sleepwalking reveals how algorithmic systems generate ‘epistemic asymmetries’ (Ruckenstein and Schüll 2017): Clinical infrastructures privilege efficiency and standardisation, whereas self‐tracking shifts interpretative responsibility to individuals. Detecting sleepwalking requires the synthesis of heterogeneous data streams—physiological signals, sociotechnical observations and narratives—and the capacity to connect them meaningfully. Epistemic authority within these systems is therefore not given but continually negotiated. Algorithmic operations, therefore, synthesise the various elements of a situation and their relationships. In this sense, algorithms can be understood as ‘affordance‐in‐figurations’ (Wettmann and Peper 2023, 237): Their capacity to produce meaning unfolds only through situated, relational practices. In clinical infrastructures, such negotiations of sleepwalking are shaped by professional routines, institutional constraints and a hierarchy of diagnostic priorities. Although video‐assisted PSG offers rich datasets, algorithmic assessment systems are optimised for SRBDs, rendering parasomnias such as sleepwalking artefacts. Here, recognition is constituted through relational practices, including attention to patients' narratives, interpretation of behavioural abnormalities and the use of video material. In self‐tracking, by contrast, this negotiation becomes a vernacular epistemic practice: Users combine digital traces with anecdotal or embodied knowledge, consulting partners or recordings to construct a personal interpretation of their sleep. This shift highlights not passive data consumption but active engagement with algorithmic uncertainty.

However, the algorithmic mapping of sleepwalking and its negotiation are shaped by epistemic inequalities. Clinical contexts possess more resources but are also constrained by institutional logics of efficiency and standardisation. Self‐tracking, in turn, offers more openness but shifts interpretative work to individuals without adequate training. In both cases, legitimate knowledge is not predetermined but actively constructed within infrastructural and social frameworks. Sleepwalking thus challenges not only the epistemic authority of algorithms but also the systems in which they operate, as the epistemic ecosystem privileges the regular and SRBDs, while illustrating how the phenomenon functions as a boundary object across medical and everyday domains. Sleepwalking disrupts this logic by challenging the notion of complete sleep or wakefulness. Consequently, algorithmic mapping must be understood not as a purely technical process but as an ongoing social negotiation unfolding across bodies, institutions and everyday life. With the case of sleepwalking, we demonstrate that algorithmic systems in healthcare do not simply fail or succeed but instead participate in ongoing processes of negotiation through which knowledge, responsibility and agency are distributed. To give sleepwalking meaning, a pluralistic epistemology that values relational knowledge, allows for uncertainty and considers the lived realities of users and experts alike is required. Algorithmic systems in the case of sleepwalking should neither be rejected nor idealised but contextualised: Their logics should be understood, their blind spots addressed and their authority and produced data situated.

## Author Contributions

**Svenja Reinhardt:** conceptualization (equal), formal analysis (equal), investigation (equal), methodology (equal), writing – original draft (equal). **Nico Wettmann:** conceptualization (equal), formal analysis (equal), investigation (equal), methodology (equal), writing – original draft (equal).

## Funding

This project has received funding from the German Research Foundation (Deutsche Forschungsgemeinschaft) under Grant 436267559.

## Ethics Statement

The research project is guided by the Code of Ethics of the German Sociological Association, in particular the principles of nonharm and informed consent. The Research and Responsibility Commission of Philipps University of Marburg has reviewed the research proposal and concluded that the project does not raise any ethical issues relevant to security research.

## Consent

The sleep laboratories and patients were presented with a data protection and research ethics concept, to which they all agreed.

## Conflicts of Interest

The authors declare no conflicts of interest.

## Data Availability

The research data used can be obtained from the corresponding author upon reasonable request.

## Endnotes

^1^
Meta‐analyses show that sleepwalking has a lifetime prevalence of 18%–29%, with some studies reporting rates of up to 40%. The current prevalence (defined as the 95th percentile within the last 12 months) is reported to be 3.8%–6.5% in children and adolescents, compared to 1.0%–2.3% in adults (Heidbreder 2020a; Stallman and Kohler 2016).

^2^
In our sample, self‐harm and aggressive behaviour against others included breaking bones (especially legs and toes), jumping out of windows, sexual aggression against other people, hitting, verbal insults and screaming.

^3^
From this standpoint, ‘the limits of the term algorithm are determined by social engagements rather than by technological or material constraints’ (Dourish 2016, 3).

^4^
In the following, it is assumed that the standard period of a person's sleep occurs at night and, therefore, ‘night’ may be used while meaning sleep.
